# Emergence of a novel lineage containing a prophage in *emm*/M3 group A *Streptococcus* associated with upsurge in invasive disease in the UK

**DOI:** 10.1099/mgen.0.000059

**Published:** 2016-06-24

**Authors:** Ali Al-Shahib, Anthony Underwood, Baharak Afshar, Claire E. Turner, Theresa Lamagni, Shiranee Sriskandan, Androulla Efstratiou

**Affiliations:** ^1^​ Disease and Informatics, 61 Colindale Avenue, Public Health England, Colindale, UK; ^2^​ Public Health England, UK; ^3^​ Imperial College London, London, UK

**Keywords:** Group A Streptococcus Prophage SpeC

## Abstract

A sudden increase in invasive Group A *Streptococcus* (iGAS) infections associated with *emm*/M3 isolates during the winter of 2008/09 prompted the initiation of enhanced surveillance in England. In order to characterise the population of *emm*/M3 GAS within the UK and determine bacterial factors that might be responsible for this upsurge, 442 *emm*/M3 isolates from cases of invasive and non-invasive infections during the period 2001–2013 were subjected to whole genome sequencing. MLST analysis differentiated *emm*/M3 isolates into three sequence types (STs): ST15, ST315 and ST406. Analysis of the whole genome SNP-based phylogeny showed that the majority of isolates from the 2008–2009 upsurge period belonged to a distinct lineage characterized by the presence of a prophage carrying the speC exotoxin and spd1 DNAase genes but loss of two other prophages considered typical of the *emm*/M3 lineage. This lineage was significantly associated with the upsurge in iGAS cases and we postulate that the upsurge could be attributed in part to expansion of this novel prophage-containing lineage within the population. The study underlines the importance of prompt genomic analysis of changes in the GAS population, providing an advanced public health warning system for newly emergent, pathogenic strains.

## Data Summary

1. The Illumina sequence reported in this paper has been deposited in the ENA Sequence Read Archive database. Accession no. ERP000535 (url - http://www.ebi.ac.uk/ena/data/view/ERP000535).
2. The ФUK-M3.1 prophage sequence ENA study accession is PRJEB13322 (http://www.ebi.ac.uk/ena/data/view/PRJEB13322)

## Impact Statement

Invasive Group A *Streptococcus* (GAS) infections cause significant mortality worldwide each year. In 2009, an unusual upsurge of iGAS infections caused by the genotype/serotype *emm*/M3 was observed in the UK. We aimed to understand the reasons behind this upsurge through whole genome sequence analysis of *emm*/M3 strains isolates between 2001 and 2013. By examining the core and accessory genomes we identified a new lineage of *emm*/M3 associated with a prophage potentially responsible for the upsurge seen in 2009. Ongoing prophage surveillance can provide early warning of proliferation of lineages causing increased incidence of severe disease. Prompt identification of such emergent lineages may permit public health interventions to be developed at an early stage.

## Introduction

Group A *Streptococcus* (GAS) has long been recognized as a human pathogen responsible for a diverse range of diseases. GAS infections cause significant morbidity and mortality globally, largely attributable to rheumatic heart disease and invasive infection. The minimum estimate, of over 500 000 deaths per year, places GAS among the major human pathogens (Carapetis *et al.,* 2005). The organism itself possesses numerous surface-associated and secreted proteins that play a key role in host–bacteria interaction such as adherence and immune evasion (Bisno* et al.,* 2003; Cunningham, 2000) and are therefore subject to strong selective pressure. M-protein is one such surface protein encoded by the* emm* gene that acts as a major virulence factor, and provides the basis for molecular typing.

**Fig. 1.**
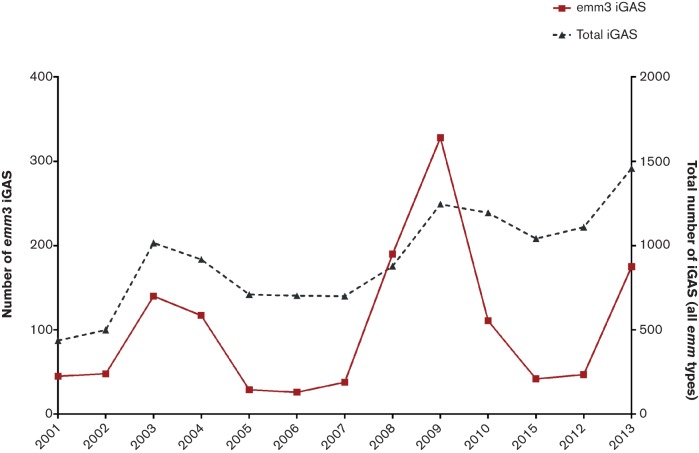
Annual number of invasive GAS cases and invasive* emm*/M3 cases identified by the national reference laboratory, UK, 2001 to –2013. The unusual increase in* emm*/M3 iGAS cases was seen from November 2008 to April 2009.

An unusual increase in invasive GAS (iGAS) infections was first reported in the UK in November 2008 (Health Protection Report, 2009) (Fig. 1). Concerns over the increased incidence and increased case fatality ratio led to initiation of enhanced surveillance for iGAS infection (Lamagni *et al.,* 2009). Assessment of more than 1200 sterile-site GAS isolates referred to the national *Streptococcus* and Diphtheria Reference Unit between January and July 2009 identified a significant increase in* emm*/M3 isolates, rising from 14 % in in the previous year to 38 % in April 2009. Such type-specific dominance had never been described in the UK and generated considerable concern given the association between* emm*/M3 and severe disease presentation (Lamagni *et al.,* 2009). There was no increase in any particular risk group but the proportion of infections in children has risen, to 22 % in comparison to 15 % in the 2003–2004 surveillance. Substantial increases in scarlet fever notifications were also documented during the upsurge period, some of which were also linked to* emm*/M3 isolates (Health Protection Report, 2008). The primary goal of this study was to investigate the observed changes in iGAS disease epidemiology in the UK in the upsurge period between November 2008 and April 2009 through bacterial whole genome sequencing of* emm*/M3 GAS isolates submitted to the reference laboratory before, during and after the upsurge.

## Methods

### Isolate collection

Microbiology laboratories in England are required to submit all sterile-site GAS isolates to the national reference laboratory for typing, and laboratories in other parts of the UK are able but not required to send isolates for typing. This UK-wide collection of isolates was used as a sampling frame to randomly select 200 GAS *emm*/M3 strains as part of this study, 160 from 2008/2009 and 40 isolates from the previous period of enhanced surveillance, January 2003 to December 2004. Sample selection was stratified by calendar quarter (grouped into 3-month periods) and age groups (<15 years, ≥16 years) which was done to ensure diversity of strains included in the study. Sixty of the 2008/2009 isolates were from clinical specimens taken during the upsurge period (November 2008 to April 2009) (Table S1, available in the online Supplementary Material). To provide more context to the data obtained and due to emerging findings suggesting the expansion of a new lineage, an additional 243 GAS *emm*/M3 isolates were included; where possible these were distributed evenly across years 2001–2013, excluding the two periods of enhanced surveillance, and from different geographical locations within the UK. In addition, four isolates from outside the UK (two from Dublin and two from Copenhagen) were also included. To set the context for* emm*/M3 isolates in the UK over longer periods of time we also sequenced two isolates from 1980 (ERS311347, ERS311348), one isolate from 1981 (ERS311349), one isolate from 1935 (NCTC 8191, ERS311351) and one isolate with an unknown date. The total number of whole genome sequenced isolates was 447.

### Whole genome sequencing

Genomic DNA extracts from the 447 GAS isolates were prepared using the Wizard Genomic DNA purification kit (Promega). Unique index-tagged libraries for each isolate were created, and pools of 96 separate libraries were sequenced using 75 bp reads on an Illumina HiSeq 2000 machine according to the manufacturer’s protocols. The index-tag sequence information was used for downstream processing to assign reads to individual isolates.

### Genomic data pipeline and analysis

#### Mapping, SNP calling and phylogenetic tree construction.

The Illumina sequence short read fastq files from all isolates were trimmed for quality by removing leading and trailing nucleotides of Phred quality score Q< 30, truncating a read if a sliding window of size 4 has a mean Q< 30, and dropping a whole read if shorter than 50 nt after trimming. The trimmed reads were then mapped onto the emm/M3 reference genome (strain MGAS315, GenBank accession no. NC_004070) using the bwa mapping tool (version 0.7.9a) (Li & Durbin, 2009). Then bwa mem was used to generate alignments in SAM format, the SAM files converted to BAM format using samtools (version 1.1) (Li *et al.*, 2009) (parameters: view –buhs) and BAM files were sorted and indexed using samtools (commands sort and index, respectively).

Candidate SNPs were identified using Genome Analysis Toolkit 2 (gatk2) (McKenna *et al.,* 2010) in UnifiedGenotyper mode. The parameters used were --isolate_ploidy: 2; --genotype_likelihoods_model: BOTH; -rf: BadCigar; mode: EMIT_ALL_SITES. With all sites omitted, the gatk2 SelectVariants method was then used to generate the Variant Call Format (VCF) files. Gubbins software (Croucher *et al.*, 2015) was used to avoid selecting possible recombination sites. Bespoke scripts written in the Python language were used to select candidate SNPs if DP (depth of coverage) was greater than 5, AD ratio (the ratio of the unfiltered count of all reads that carried that specific allele compared with other REF and ALT alleles in that site) was greater than 0.8, MQ (mapping quality) was greater than 30 and no more than 0.05 of reads mapping at the position possessed a mapping quality of 0 (MQ0). Heterozygous and SNP positions filtered out by the metrics listed were replaced with the character ‘N’. For each isolate, output was directed to a serialized Python pickle file. Pickle files were then combined to generate a single multiple alignment concatenated fasta file containing filtered SNPs with the maximum proportion of Ns to accept in any column in the alignment set at 0.1. The script also excluded SNPs within prophage elements based on the MGAS315 genome prophage coordinates [aken from MGAS315 enank file (http://www.ncbi.nlsm.nih.gov/nuccore/NC_004070.1)]. Maximum-likelihood (ML) phylogenetic trees were then reconstructed using RAxML (Stamatakis *et al.,* 2006). [Initial phylogenetic trees were reconstructed using the mega phylogenetic tree analysis tool (Kumar *et al.,* 2008).]

#### *De novo* assembly.

Reads were assembled using velvet (version 1.2.10) (Zerbino *et al.,* 2008). The velvet *shuffleSequences_fastq.pl* script was used to produce a shuffled FASTQ file to become the input for VelvetOptimiser (version 2.1.9) (Gladman *et al.,* 2012) to optimize the cumulative rank for *N*_50_ with minimum and maximum Kmer lengths of 55 and 75, respectively (*–s 55 –e 75 –f '-shortPaired'*). The resulting contigs were used to extract the MLST type of each isolate by comparing it with the MLST *Streptococcus pyogenes* database (http://spyogenes.mlst.net) using blast+ (Camacho *et al.,* 2009). The MLST types were mapped onto the ML tree.

#### Accessory genome investigation.

To investigate the phage content of each isolate, reads were mapped in a local alignment mode using Bowtie 2 (http://bowtie-bio.sourceforge.net/bowtie2/index.shtml) against a set of all the identified *S. pyogenes* prophages available in GenBank (total of 53 prophages, Table S2) to generate a sequence alignment/map (SAM) file. After converting the SAM file to BAM file format, the BAM file was used to generate a variant-calling file (VCF) using the Samtools mpileup (Li *et al.,* 2009) algorithm with default settings. Base polymorphisms were detected using an in-house Python script which parsed the VCF file line-by-line to determine the base-call at each nucleotide position. This list was filtered if the SNP had coverage five or more reads, frequency of polymorphic bases was ≥ 80 % and the overall quality of the variant call (i.e. base mapping) was ≥  25 phred score. The algorithm then generates an overall identity score for each prophage sequence. Isolates showing over 90 % nucleotides identified over 100 % length of the prophage sequence were considered as present.

## Results

### Isolates and their sequence types

**Fig. 2.**
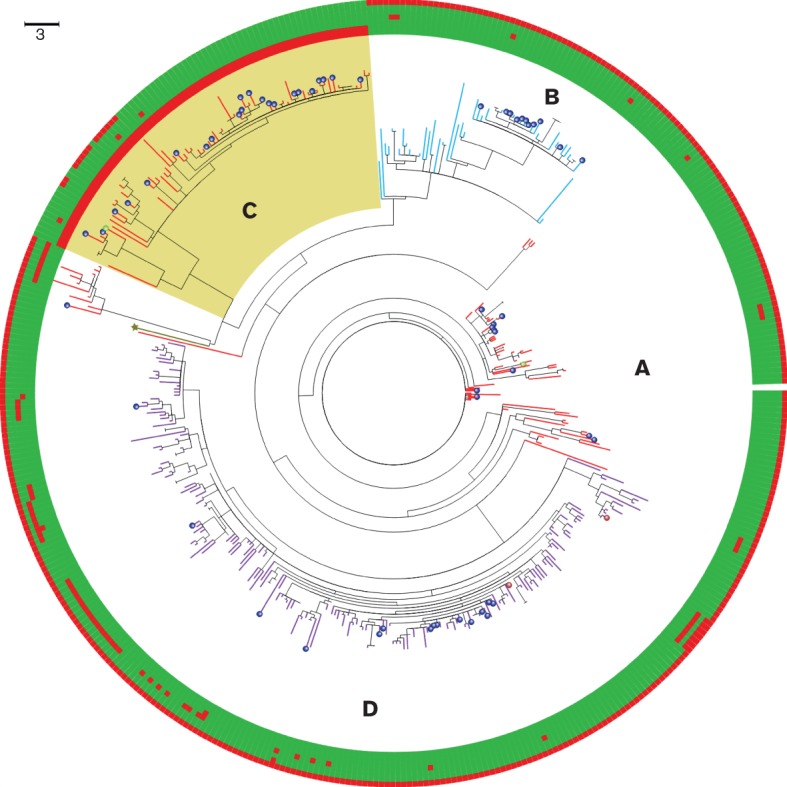
Annotated circular ML SNP tree with prophage presence/absence.Tree branches are coloured according to STs: red, ST15 (Lineages A and C); light blue, ST406 (Lineage B); and purple, ST315 (Lineage D). The yellow shaded region contains the isolates that mostly contain the ФUK-M3.1 prophage (Lineage C), except for 13 isolates. Isolates from the upsurge period are labelled with a dark blue sphere at the tip of the branches. The two Dublin isolates are coloured with a green sphere and the two Copenhagen isolates are coloured with a brown sphere. The outer circle contains five rings that indicate the presence (green) and absence (red) of all MGAS315 genome prophages for each isolate. Each ring is aligned with its node in the tree. The rings are presented in the following order, beginning from the closest ring to the tree (innermost to outermost): prophages Ф315.1, Ф315.2, Ф315.3, Ф315.4, Ф315.5, Ф315.6, ФUK-M3.1. All isolates shown on the tree contain the Ф315.1 and Ф315.2 prophages except those in Lineage C. In addition, phage ФUK-M3.1 is only seen in Lineage C. The reference strain MGAS315 branch is coloured with a dark green star at the tip of the branch. Eight isolates [the 1935 isolate (NCTC8191), three 1980s isolates and four other isolates] were excluded from the tree based on having large branch distances. Bar, approximately 3 SNPs.

To determine if* emm*/M3 isolates from the iGAS upsurge period (November 2008 to April 2009) were distinct in any way, the genomes of 447* emm*/M3 isolates, including 60 from the upsurge period, were sequenced. High-quality SNPs derived by mapping to reference sequence MGAS315 (*emm*/M3) were used to generate a ML phylogenetic tree (Fig. 2). A total of 3184 SNPs were found amongst all isolates. MLST data extracted *in silico* from the contigs derived by the *de novo* assemblies differentiated all isolates into one of three sequence types (STs): ST315 (211, 48 %), ST15 (181, 40 %) or ST406 (55, 12 %). The isolates of type ST315 and ST406 were confined to single clearly differentiated clonal lineages whereas ST15 was found in multiple ST15-specific lineages, including those designated Lineages A and C (Fig. 2). ST315 isolates (Lineage D) were predominant from 2001 to 2006 (ST315: 76 %, ST406: 14 %, ST15: 10 %). Isolates from the period of the upsurge were observed in all lineages, excluding the possibility that upsurge cases could be wholly attributed to a single lineage (Fig. 2).

### Pan genome analysis: association of prophage ФUK-M3.1-carrying lineage with upsurge

To determine whether changes in the pan genome occurred during the upsurge period, we compared the ratios of all genes within the isolates in the upsurge period with all other isolates in this study and identified those observed more often than expected during the upsurge period.

**Fig. 3.**
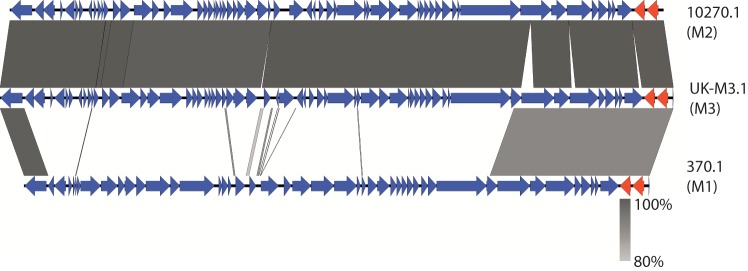
A blast comparison between prophage Ф10270.1 (from MGAS10270* emm*/M2 genome), prophage ФUK-M3.1 (from *de novo* sequenced* emm*/M3 genome ERS024021) and prophage Ф370.1 (from MGAS370* emm*/M1 genome). Red arrows from left to right represent the virulent genes *speC* and *spd1* respectively. This was drawn using the Easyfig tool (Sullivan *et al.,* 2011).

A total of 23 genes were significantly over-represented (<0.05, *t*-test) amongst isolates from the upsurge period compared with those outside this period. All 23 genes were located within a prophage and were limited to a particular lineage, designated Lineage C (Fig. 2). This prophage, hereafter named ФUK-M3.1, comprised 63 genes that included the gene encoding streptococcal pyrogenic exotoxin C (*speC*) and the prophage-encoded DNase (*spd1*). Prophage ФUK-M3.1 was 44  kb in length with DNA G+C content of 38 mol%. The assembled prophage sequence (ENA study accession: PRJEB13322, derived from *de novo* sequenced* emm*/M3 genome ERS024021) shared ~97 % DNA identity over 100 % length with other *speC*/*spd1*-associated prophages: Ф10270.1 (*emm*/M2), Ф10750.1 (*emm*/M4) and Ф9429.3 (*emm*/M12) (Beres & Musser, 2007) but only ~30 % DNA identity with the* emm*/M1 *speC-* and *spd1*-containing prophage Ф370.1 (Ferretti *et al.*, 2001) (Fig. 3).

**Fig. 4.**
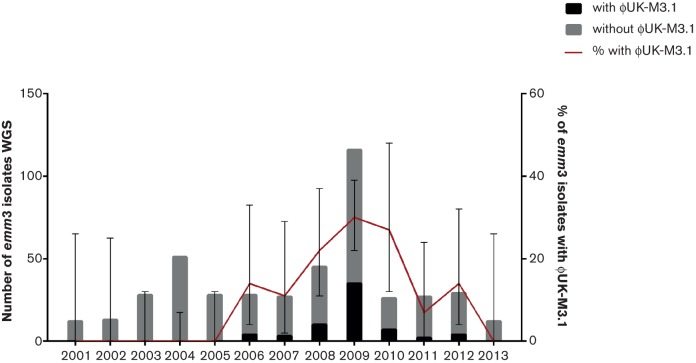
Number of* emm*/M3 cases analysed by whole genome sequencing (442) between 2001 and 2013, with (shown in black) and without (shown in grey) ΦUK-M3.1 prophage. Also indicated are the exact bionomial 95% confidence intervals for each year. The upsurge period was from November 2008 to April 2009. The number of *emm*/M3 isolates with the ΦUK-M3.1 prophage correlated with the upsurge period and decreased following the upsurge period.

Lineage C comprised 78 isolates, 65 of which carried the complete ФUK-M3.1 prophage, while the remainder either did not carry it or contained only an incomplete prophage (without the *speC* or the *spd1* genes). Of these 65 isolates, 46 (70 %) were from invasive cases and the remainder from non-invasive cases. Although the isolates sequenced within Lineage C were derived from clinical isolates taken over a period of 10 years, one-third (22/65) of the ФUK-M3.1-containing isolates were from the 6 month upsurge period. These ФUK-M3.1-containing isolates accounted for 22 of the 60 upsurge cases included in the study. Although the numbers of *emm*3 isolates varied across the study period, it is noteworthy that isolates carrying prophage ФUK-M3.1 were not observed until 2006 and diminished in subsequent years (Fig. 4).

Isolates from Lineage C were significantly over-represented [χ^2^ (1 d.f.)  =  17.77;  <0.0001] in the upsurge period compared with any other lineage (Table 1). In contrast, isolates from other lineages, predominantly Lineage D, were significantly under-represented (<0.0026) in the upsurge period compared withother lineages. No significant differences were identified between the other lineages.

**Table 1.** Number of isolates in Lineage C during the upsurge period (November 2008 to April 2009) and before and after the upsurge period (from 1935, 1980–1981 , 2001–2013 except November 2008 to April 2009) The percentage of isolates is given in parentheses. Isolates from Lineage C were significantly over-represented (< 0.0001) in the upsurge period compared with any other lineage.

	Upsurge period	Non-upsurge period	Total
Lineage C	22 (36.6 %)	56 (14.5 %)	78
Other ineages	38	331	369
Total	60 (100 %)	387 (100 %)	447

### Bacteriophage content of isolates: absence of Ф315.1 and Ф315.2 in Lineage C

Reads from the genomes of each isolate sequenced were mapped against 53 GAS prophages (Table S2). Three major findings were revealed. Firstly, none of the isolates in Lineage C that contained the ФUK-M3.1 prophage contained the Ф315.1 and Ф315.2 prophages (Fig. 2) that are typical of* emm*/M3 *S. pyogenes*. Secondly, with reference to the SNP phylogeny (Fig. 2), all non-Lineage C isolates contained the Ф315.1 and Ф315.2 prophages, consistent with reports that show the presence of Ф315.1 and Ф315.2 prophages in* emm*/M3 isolates across the globe (Beres *et al.*, 2004; Meisal *et al.*, 1998; Sharkawy *et al.,* 2002). Thirdly, 13 isolates in Lineage C contained neither the Ф315.1, the Ф315.2 nor the ФUK-M3.1 prophage, which suggested that, although isolates in Lineage C shared a same common ancestor, a few had subsequently lost or have an incomplete ФUK-M3.1 prophage. All isolates in Lineage C also carried the other typical *emm*/M3 prophages: Ф315.3 (associated with DNAse *spd*4), Ф315.5 (associated with superantigen *speA*) and Ф315.6 (associated with DNAse sdn); and 75/78 carried Ф315.4 (associated with superantigen *speK* and phospholipase *slaA*).

### Non-UK and older isolates

Of the four non-UK isolates (two from Dublin isolated in 2006 and 2012 and two from Copenhagen isolated in 2007 and 2008) sequenced in this study, one strain from Dublin (accession no. ERS311234 isolated in 2006), which contained the ФUK-M3.1 prophage, was associated with Lineage C while the other three were in the other lineages in the phylogeny (Lineages A, B and D in Fig. 2). Data from 86* emm*/M3 GAS genomes collected from Ontario, Canada, between 2003 and 2009 taken from Shea *et al.* (2011) were analysed and incorporated into the phylogeny (data not shown). This revealed that six strains (accession nos. SRR125478, SRR125479, SRR125450, SRR125480, SRR125449, SRR125474) from the Ontario collection, isolated from 2002 to 2009) fell within Lineage C and also contained the ФUK-M3.1 prophage, while others were distributed across the other lineages.

As part of this study, we also sequenced three isolates from the 1980s and one isolate from 1935 (NCTC 8191, ERS311351). The 1980s isolates did not contain the ФUK-M3.1 prophage and belonged to a separate lineage. They did, however, contain the Ф315.1 and Ф315.2 prophages. The 1935 isolate contained the ФUK-M3.1 prophage, whilst prophages Ф315.1 and Ф315.2 were missing. Interestingly, this isolate did not fall within Lineage C associated with the upsurge but instead belonged to another lineage derived from the common ancestor of ST15 strains.

## Discussion

GAS* emm*/M3 strains are associated with severe infections and are associated with a higher likelihood of streptococcal toxic shock syndrome, necrotizing fasciitis and death in some patients (Banks *et al.,* 2002). The main objectives of this study were to determine whether there was a pathogen-encoded factor or factors that may have been responsible for the upsurge in* emm*/M3 isolates observed in late 2008/early 2009 causing invasive GAS disease in England. Based on whole genome phylogeny we identified and characterized a new clonal lineage of* emm*/M3 GAS that was not present in detectable numbers in the collection examined before 2006. This lineage (Lineage C) was significantly associated with the upsurge period, and accounted for approximately one-third of cases within the upsurge. Furthermore, accessory genome analysis demonstrated that this lineage had gained a novel bacteriophage (ФUK-M3.1) containing the genes *speC* and *spd1* but lost two typical* emm*/M3 prophages: Ф315.1 and Ф315.2.

We considered the possibility that the absence of prophages Ф315.1 and Ф315.2 typically found among *emm*/*M3* isolates might be relevant to the transient success of Lineage C. Prophage Ф315.1 has a different insertion site from Ф315.2 and does not contain any known virulence factors; however, it is sited within the single CRISPR locus found within the *emm*/*M3* genome. The absence of Ф315.1 among strains of Lineage C could potentially restore the CRISPR locus and may influence not only susceptibility to new DNA uptake, but also expression of virulence factors (Nozawa *et al.,* 2011). Prophage Ф315.2 is a T12-like prophage that includes the superantigen* ssa* gene and is inserted at the predicted T12_att_ site. While the presence of phage-encoded superantigen genes is considered likely to confer advantage to GAS, it may be that acquisition of additional phages that encode alternative possibly more potent superantigens or other virulence factors compensates for this loss.

The proportion of *emm* types circulating in a population has been shown to vary often in a cyclical nature and periodic surges in specific *emm* types have previously been linked to the emergence of distinct clades. These clades have expanded and apparently replaced earlier lineages, for example the emergence of the modern *emm*/M1 (Nasser *et al.,* 2014) and, more recently, acapsular *emm*/M89 through recombination-related remodelling of the genome (Turner *et al.,* 2015). In this study we report an increase in the proportion of *emm*/M3 strains within the UK population and show that this was at least in part due to a lineage that was recently introduced into the UK. This lineage was distinguished by having a phage containing the *speC* and *spd* genes, a combination not seen in any of the phages that are commonly observed in *emm*/*M3* strains. Unlike the rise of the acapsular *emm*/M89 lineage, which has been sustained, the 2009 rise in Lineage C was short-lived; a further rise in 2013 in *emm*/M3 was not associated with the same lineage or phage combinations. An apparent overall rise in iGAS in 2013 was accounted for by rises in several *emm* types including *emm*/M1. It would appear that *S. pyogenes* lineages can adopt a range of strategies to expand within a population, resulting in changes that are of varying durability.

Bacteriophages comprise 12 % of the published* emm*/M3 GAS genome MGAS315 and three of the four prophages found in MGAS315 are associated with at least one extracellular virulence factor including the superantigenic toxins *ssa*,* speK* and *speA* and the phospholipase *slaA* (Beres *et al.,* 2002). Infection is likely to have accounted for the initial acquisition of ФUK-M3.1 by the common ancestor of the 78 isolates within Lineage C. However, in 13 of the isolates, ФUK-M3.1 was either absent or incomplete, perhaps through excision of the prophage. Both the *speC* and the *spd1* genes can be associated with many different prophages that have been identified in the published* emm*/M1,* emm*/M2,* emm*/M4,* emm*/M5,* emm*/M6,* emm*/M12,* emm*/M18 and* emm*/M28 genomes. A blast comparison revealed that ФUK-M3.1 is similar to the prophage identified in the published* emm*/M2,* emm*/M4 and* emm*/M12 GAS genomes, which suggested that if the prophage was acquired by a horizontal acquisition event the donor may have been one of these M types, although there is evidence that GAS share their phage pool with other species (Musser *et al.,* 1991). The presence of a prophage containing the *speC* and *spd1* genes in* emm*/M3 GAS has been detected, albeit rarely, in some countries outside the UK (Meisal *et al.,* 1998; Musser *et al.,* 1991; Sharkawy *et al.,* 2002) and in our study in a single 1930s isolate, but is most commonly absent from *emm*/M3 isolates (Commons *et al.,* 2008; [Bibr R13][Bibr R29]). The ФUK-M3.1 prophage was not detected in any contemporary* emm*/M3 isolates from our study prior to 2006 (Fig. 4). Therefore, we speculate that the lineage associated with the upsurge may have arisen by introduction of this strain into the UK from abroad, and resulted in a short-lived upsurge in severe disease phenotypes associated with GAS infection. To support this hypothesis, isolates collected from the study in Ontario, Canada (Shea *et al.,* 2011), between 2003 and 2009 and two collected from Dublin (2006, 2012) and two collected from Copenhagen (2006, 2009) in this study were mapped onto the phylogeny. Six isolates from the 86 isolates in Ontario were found within Lineage C. This suggested that Lineage C was not exclusive to the UK and was found in other populations. The prophage Ф315.1 and the* ssa*associated prophage Ф315.2 were absent from the isolates in Lineage C. This was surprising as prophages Ф315.1 and Ф315.2 were present in all other* emm*/M3 isolates sequenced and many of those seen in other areas worldwide (Commons *et al.*, 2008; Ferretti *et al.,* 2001; Nozawa *et al.,* 2011). We propose that the acquisition of ФUK-M3.1 and loss of Ф315.1 and Ф315.2 occurred independently rather than replacement of one with the other given that the integrated prophage hybrid sites positions *attL* and *attR* in the genomes are dissimilar, although we cannot exclude biological interference between the three prophages. The overarching question arising from such studies remains the reason for the association of the presence of ФUK-M3.1 and other phages with the success of dominant lineages. Superantigens, such as *speC*, are hypothesized to undermine host immunity potentially through T cell anergy, although direct evidence for this in the clinical setting is lacking (Llewelyn & Cohen, 2002). From an evolutionary standpoint, any advantage to the bacterium is likely to impact more on pharyngeal infection and transmission than invasiveness. Evidence from animal models supports a role for prophage-encoded superantigens in pharyngeal infection (Kasper *et al.,* 2014; Virtaneva *et al.,* 2005); however, whether T cell-related immunoparesis is important is unclear.

## Conclusions

The upsurge in invasive *emm*/M3 GAS infections in England in 2008/2009 was associated with the emergence of a novel lineage of* emm*/M3 GAS isolates within the population. Decreased population immunity to this novel genetic variant coupled with biological advantage conferred by carriage of the *speC*/*spd1*-associated prophage ФUK-M3.1 may have potentially permitted expansion of this lineage throughout the UK, although we cannot exclude the role of other lineage-specific molecular changes. Acquisition of prophages may be a common feature of newly or rapidly emergent streptococcal lineages, but may only partly explain the success of such lineages. The expansion of *emm*/M3 lineage C containing the ФUK-M3.1 prophage does not appear to have been as enduring as the expansion observed for the modern *emm*/M1 and novel *emm*/M89 lineages in the UK and we have not detected ФUK-M3.1 in isolates from 2014–2015 (our unpublished data). Longitudinal molecular–epidemiological surveillance of prophage and toxin gene content within distinct GAS lineages could provide greater understanding of the contribution that such prophages make to periodic changes that occur in both upper respiratory tract and iGAS disease abundance. Furthermore, such surveillance, if applied to upper respiratory tract isolates, could provide early warning of lineages that may have a propensity for rapid expansion, thus facilitating potential public health interventions.
