# Corrigendum: Emergence of a novel lineage containing a prophage in *emm*/M3 group A *Streptococcus* associated with upsurge in invasive disease in the UK

**DOI:** 10.1099/mgen.0.000097

**Published:** 2016-11-30

**Authors:** Ali Al-Shahib, Anthony Underwood, Baharak Afshar, Claire E. Turner, Theresa Lamagni​, Shiranee Sriskandan, Androulla Efstratiou

The following acknowledgements were omitted from the published article:

## Acknowledgements

The authors would like to thank Juliana Coelho (PHE) and Sarah Philips for their support in extracting DNA for *emm* subtyping and a proportion of isolates for sequencing, George Kafatos for help in the statistical analysis of the data and Michel Doumith for using his tool to analyse the Pan genome. They would also like to thank the core sequencing and informatics teams at the Sanger Institute for their assistance and The Wellcome Trust for its support of the Sanger Institute Pathogen Genomics and Biology groups. They also thank Matthew Holden and Julian Parkhill for their support of the project at the Sanger Institute. The authors thank the National Institute of Health Research for Health Protection Research (NIHR CHPR) programme for funding this study (project number: 106846/107513). Shiranee Sriskandan acknowledges the Imperial College NIHR Biomedical Research Centre.

